# ‘Five hours to sort out your life’: qualitative study of the experiences of university students who access mental health support

**DOI:** 10.1192/bjo.2021.947

**Published:** 2021-06-24

**Authors:** Phoebe Barnett, Laura-Louise Arundell, Hannah Matthews, Rob Saunders, Stephen Pilling

**Affiliations:** Centre for Outcomes Research and Effectiveness, Research Department of Clinical, Educational, and Health Psychology, University College London, UK; Centre for Outcomes Research and Effectiveness, Research Department of Clinical, Educational, and Health Psychology, University College London, UK; and National Collaborating Centre for Mental Health, Royal College of Psychiatrists, UK; Health Economics and Outcomes Research, UK; Centre for Outcomes Research and Effectiveness, Research Department of Clinical, Educational, and Health Psychology, University College London, UK; Centre for Outcomes Research and Effectiveness, Research Department of Clinical, Educational, and Health Psychology, University College London, UK; National Collaborating Centre for Mental Health, Royal College of Psychiatrists, UK; and Camden and Islington NHS Foundation Trust, UK

**Keywords:** University, mental health, psychological interventions, thematic analysis, student support

## Abstract

**Background:**

Previous qualitative research suggests that university students feel that current service provision does not meet their needs. Exploring the reasons for this may help to promote service change, encourage the uptake of care, improve outcomes and increase satisfaction within university services.

**Aims:**

This study aimed to improve the understanding of how students experience the process of accessing and using mental health support, barriers and facilitators to treatment, and how students would adapt provision to improve experiences.

**Method:**

Semi-structured interviews were conducted with 16 full-time students who had used mental health services at university. Data were analysed using thematic analysis.

**Results:**

Five higher-order themes were identified: personalisation and informed choice, simplifying the process, feeling abandoned ignored or invisible, stigma, and superiority of private and external services. Sixteen subthemes were identified within these themes.

**Conclusions:**

Findings indicate that access to mental health support should be simplified, with collaboration across university and external health and care services, to prevent students feeling lost or abandoned when seeking care. An inclusive approach to support access and provision of services for all presentations of mental health problems should be developed.

The well-being of university students is an international concern.^1,2^ Although university settings could provide important opportunities to prevent and treat mental health problems,^3^ current evidence suggests that this is not the case. A UK study reported that overall psychological distress does not fall below pre-admission levels at any point during university,^4^ and similar distress levels have been observed internationally.^5^

## Continuing prevalence of mental health disorders in university students

Several factors could contribute to the continuing prevalence of mental health problems in students. For example, studies suggest that students feel existing services are inappropriate for their needs,^6–8^ and that collaboration between staff responsible for student mental health with those responsible for education is uncommon.^9^ This might prevent some students from seeking help, with only half of students who experience significant levels of anxiety or depressive symptoms going on to contact professionals.^7^ The roots of these hesitations are likely multifaceted and may include stigma,^7,10,11^ lack of understanding of symptoms^7,8^ and confidentiality concerns.^7^ Furthermore, young people express fears that treatment providers will be judgemental, lack insight into the experience of young people or be too busy to listen.^7,11^

## Adaptation of current service delivery

Research also suggests that students who do seek help report dissatisfaction with available services,^12^ and young adults disengage from treatment more than other age groups.^11,13^ Involving students in service design could positively influence aspects of treatment provision currently concerning students, such as lack of treatment choice^14,15^ or time available alongside studies.^16^ Participatory design methods have proven effective in the cultural adaptation of university support,^17^ suggesting that similar methods could be usefully integrated into design of services for all university students. Although previous efforts to make interventions more ‘student friendly’ have failed to demonstrate benefits,^18^ a service delivery model co-produced with students may fair better than adaptations developed by professionals alone. Such an approach to restructuring current support systems, drawing on student experiences and expectations, may better identify, assess and respond to student needs. This could in turn encourage service uptake and maximise on a unique opportunity to improve well-being in young people at a crucial point in their lives.

## Aims

To better understand how service provision could be improved, a qualitative approach is necessary, aiming to update the literature in this area^15^ and further enrich information about current experiences of university mental health provision. This study aims to conduct in-depth interviews with students from a variety of backgrounds to gain an understanding of how students experience the process of accessing and using in-house mental health support services at a specific institution; the barriers and facilitators to treatment, and reasons behind negative or positive experiences; and student recommendations for further service development.

## Method

### Study design and theoretical perspective

An individual semi-structured interview method was employed to understand experiences of support received for mental health problems at university. A further confirmatory focus group was undertaken to validate the identified themes. This research took a realist approach to research design and interpretation, with specific interest in experiences as reported by participants in the context of a single UK university. As a result, conceptualisations of experience remained close to the data rather than focusing on higher-order notions of semantic meaning.

### Ethical approval and informed consent

The authors assert that all procedures contributing to this work comply with the ethical standards of the relevant national and institutional committees on human experimentation and with the Helsinki Declaration of 1975, as revised in 2008. All procedures involving human patients were approved by the University College London Ethics Committee (reference number 14643/001). Informed written consent was obtained electronically from each participant before participation in the semi-structured interview. Further consent was sought for focus group participation.

### Participants

Students (*N* = 16) who had accessed any mental health support services at the university (including university-provided well-being, counselling and psychiatric services) were recruited. We aimed to recruit a diverse range of participants regarding age, gender, ethnic group, student status (undergraduate/postgraduate), subject studied and reported mental health problems, to provide a range of background experiences that could affect access to and experience of treatment.^19^ Participants who responded to a university-wide mental health survey^20^ stating they had used university mental health services, studied full-time and consented to contact regarding further research participation opportunities were eligible for participation. A purposive sample was contacted via email with an invitation to participate; however, because of a lack of response from male participants, the final sample was limited in its over-representation of female students. We were also unable to recruit any participants who identified as being non-binary, further limiting the sample. Participant's characteristics are displayed in Table 1. Focus group participation was open to all participants from the interview stage; all agreed to further contact regarding this and were emailed an invitation. Five participants responded and attended the focus group, consisting of three women and two men. Four were undergraduates and one was a postgraduate student. All were from the UK; three were White British, one was from an ‘other Asian’ background and one was of mixed ethnicity.

**Table 1:** Interview participant characteristics

Variable		*N* = 16	%		*N* = 16	%
Gender	Female	13	81%	Male	2	19%
Age, years	20–24	9	56%	25–27	7	44%
Ethnicity	White British	5	31%	White non-British	5	31%
	Mixed White	1	6%	Chinese	1	6%
	Other Asian	3	19%	Other mixed background	1	6%
Sexual orientation	Heterosexual	8	50%	Homosexual	1	6%
	Bisexual	2	13%	Other	1	6%
	Prefer not to say	4	25%			
Religious belief	No religion	9	56%	Muslim	1	6%
	Christian	2	13%	Spiritual	1	6%
	Buddhist	1	6%	Prefer not to say	2	13%
Relationship status	Relationship, not cohabiting	3	19%	Single	10	62%
	Cohabiting	3	19%			
Fee status	Home	7	44%	European Union	5	31%
	Overseas	4	25%			
Degree level	Undergraduate	10	63%	Postgraduate taught	3	19%
	Postgraduate research	3	19%			
Degree subject	English literature	2	13%	Language and international studies	2	13%
	Psychological sciences and related disciplines	2	13%	Bioscience	2	13%
	Medicine	2	13%	Computer science	1	6%
	History	1	6%	Philosophy	1	6%
	Information studies	1	6%	Combined arts and science degree	1	6%
	Prefer not to say	1	6%			
Year of study	First	6	38%	Second	5	31%
	Third	3	19%	Fourth	1	6%
	Fifth	1	6%			
Accommodation type	Private sector halls	1	6%	Parental home	2	13%
	Other rented accommodation	8	50%	Prefer not to say	5	31%
Year started degree	2013	1	6%	2015	2	13%
	2017	4	25%	2018	4	25%
	2019	5	31%			
Mental health conditions reported	Generalised anxiety disorder	8	50%	Depression	6	38%
	Bipolar disorder	2	13%	Eating disorder	3	19%
	Panic disorder	4	25%	Obsessive–compulsive disorder	2	13%
	Post-traumatic stress disorder	1	6%	Social anxiety disorder	1	6%
	Borderline personality disorder	1	6%	Autism spectrum disorder	3	19%
Treatment(s) currently utilised	Therapy, counselling or coaching	16	100%	Medication	8	50%
Support providers in contact with	General practitioner	9	56%	Mental health professional	16	100%
	University well-being services	16	100%	Telephone support	5	31%

### Setting

This study was conducted at an inner-city UK university. The university has a highly diverse student population and a large proportion of international students.

### Materials

Semi-structured interviews allowed students to freely express themselves and for new ideas to emerge, as well as facilitating a conversational focus on experiences of specific aspects of treatment, providing comparison across participants. The interview schedule was developed considering previous research^15,21^ into potential areas of difficulty in university mental health support delivery, and aimed to explore experiences of mental health support, prior expectations of what support would be available at university and how this compared with reality, and recommendations for improvement. The interview schedule was piloted with five students (who did not participate in the main interview study) with experience of mental health problems to ensure the correct language and topics were covered, so as to minimise distress and maximise relevance. Details of the interview schedule and resulting adaptations are available in Supplementary Appendix 1 available at https://doi.org/10.1192/bjo.2021.947.

### Procedure

#### Interview

First, semi-structured, individual telephone interviews were conducted with participants by one researcher (P.B.). Interviews continued until data saturation (when the ability to obtain additional new information had been attained and further coding was no longer feasible).^22^ Interviews were audio-recorded and transcribed verbatim, and lasted 36 min on average. Participants were paid £15 for attendance.

#### Focus group

A video-conference focus group with five students (who had also participated in the interview stage) allowed themes established through thematic analysis of interview transcripts to be discussed. Visual presentations of each theme (and relevant subthemes) were presented in a secure videoconferencing program, and time was given to provide suggestions for modifications or additional information. The facilitator (P.B.) ensured that all participants had an opportunity to speak, and additional communication via email was encouraged for any further thoughts that participants may have felt uncomfortable raising in a group context. However, no additional comments were received after the focus group. This focus group lasted 46 min and participants were compensated £10 for attendance. The use of individual interviews followed by a focus group provided triangulation of the data through ‘member checking’,^23^ the provision of different perspectives that complement each other.^23,24^ The interviews and focus group were conducted electronically to follow social-distancing guidelines imposed during the COVID-19 pandemic, and were kept informal to maximise alliance between the researcher and participants.

### Data analysis

A thematic analysis was conducted on interview transcripts, as it was deemed the most appropriate method because of its flexibility in theoretical stance.^25,26^ NVivo version 12 for windows (QSR international) software was used to facilitate this process. The analysis took an exploratory, inductive approach to capture emergent experiences of seeking mental health support at the university. However, interview questions were research-based, and piloted before the start of the study; therefore, the analysis was likely affected both by researcher theory and epistemological position.^26^ One researcher (P.B.) first read each transcript independently, highlighting initial themes emerging from the data. Next, codes were organised into higher-order themes that represented important aspects of experience.^25^ Themes were reviewed by P.B. and L.-L.A. separately, and differences in themes identified from a sample of the data were discussed, leading to developments to the original codes. A hierarchical thematic framework emerged as data analysis progressed. Focus group analysis was conducted after the first review of themes, and provided additional context and validation of the generated themes, allowing the resulting framework to stay grounded in the experiences of participants. The final coding structure was discussed and agreed upon by all authors. Transcripts were re-coded according to the finalised framework by P.B.

## Results

**Fig. 1:**
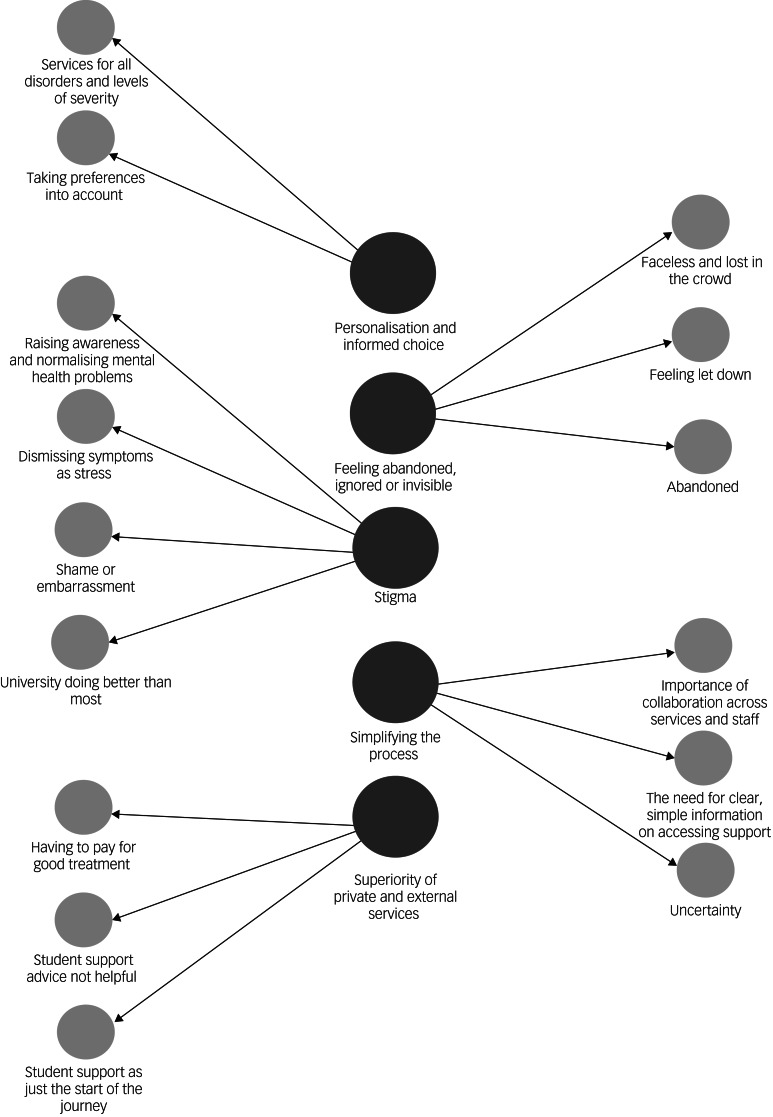
First- and second-order themes.

Five themes and 15 subthemes were identified and are represented in Figure 1. A more detailed description with supporting quotations are available in Supplementary Appendices 2–6.

### Personalisation and informed choice

Personalisation and informed choice was highlighted as an important aspect of mental health support by all 16 interviewees. Students spoke of wanting support that was appropriate for all students needing help, regardless of the complexity or severity of their problems, preventing any student from feeling excluded:

‘It comes across as psychological services for people with diagnosed conditions or specific mental health conditions. So maybe just having more like clarity in the sense that like it can be about anything, even if it's just like a difficult time’ Participant 8.

‘I find that it revolves a lot around anxiety, depression and that but they don't really talk about eating disorders, for example, which are really present in [the city] and our age … ’ Participant 13.

Discussions around choice included choice of treatment, provider and appointment time:

‘I kind of asked to see a CBT [cognitive–behavioural therapy] … but the admin say [sic] … that whether I get referred to have CBT is up to my psychiatrist’ Participant 14.

The feeling that sometimes change, personalisation or choice could not be requested because of a sense that ‘anything is better than nothing’ was also frequently mentioned:

‘For me, it was like a pressure to feel like I should be grateful for it. Like, given the state of the NHS [National Health Service], the underfunding of mental health services and a long waiting list with those, then just the fact that my university was offering something. I felt like, even though it was only six 15 min sessions, which is like 5 hours to sort out your life, I still felt like I had to be grateful that I'd been seen’ Focus group.

### Simplifying the process

Despite wanting more within-service treatment options, students also described needing a simplified process to support initial access. Students mentioned that more collaboration, both between mental health support and academic staff, as well as between different support services, may reduce confusion. In particular, students were keen for training for academic staff to better equip them to help students navigate the complexities of the support system:

‘I think it's [training] really important because … they might be the only contacts that a student is actually having … it's not an easy thing to be like “Oh I'm struggling and I need some help”’ Participant 9.

Collaboration within available mental health support services (including the student well-being service, counselling services and disability services) was also considered essential because of the current disjointed, confusing framework for care. Less overwhelming information and a single route for access was deemed an important priority for services, although students also discussed the importance of continuing support throughout referral, if needed:

‘They really need to like make a service that's support for everyone under one roof. Because right now, there's like student counselling services, which is different to student psychological and that's really confusing. Because surely they should be under one umbrella and then you can just send them to different people. Why are they two separate names? It just doesn't make sense’ Focus group.

Many students felt ‘lucky’ to have experienced mental health problems before university, as the lessons learnt through this experience, rather than university-based information, had provided the knowledge and skills needed to access support. Students with less experience described uncertainty over what to expect:

‘I have a privilege in that I know how to advocate for myself because I've done it before and I know how to be pushy and I don't feel like embarrassed about doing that’ Participant 9.

‘I had no idea who this person was and what I was supposed to speak about with them, if it was coursework, anxiety or personal issues’ Participant 10.

### Feeling abandoned, ignored or invisible

Students described feeling insignificant amongst such a large student body, and felt that making aspects of the university more personal may encourage students to ask for help, instead of going unnoticed:

‘A lot of people sort of slip through the net. … I was sort of within halls last year and I think that was probably the main sort of place that felt any kind of community, really. So yeah, particularly if someone wasn't…it'd be very easy for these things to kind of get missed.’ Participant 4.

Extensive bureaucratic tasks before being able to speak to someone exacerbated this, and was highlighted as particularly difficult when seeking help with significant levels of debilitating symptoms. The benefits of a more welcoming environment and how this could promote positive experiences were described by a few students:

‘But I felt that before I could actually speak to like a real person, I had to go through so much, filling in forms and sending emails and yeah, like months, I'm talking about months and months’ Participant 11.

‘The lady who was on the front desk at SPS [Student support services], she's lovely, she's really friendly and very helpful, really approachable and like very kind and understanding’ Participant 9.

However, stories of being ‘let down’ by services, either through getting lost in the system, being left waiting in crisis, or having sessions stopped, were frequent:

‘I feel like she [the therapist] just didn't manage to keep her promise’ Participant 1.

Despite this, students acknowledged the difficulties in providing support for such a large student body, particularly those who had experienced National Health Service waiting lists:

‘I know that in general waiting lists for counselling or psychological service is quite long …, so I don't know if 1 month is good. But to me I think it's really good … I thought it was going to be like a year or something’ Participant 14.

### Stigma

Stigma was a topic raised by the interviewer. In response, students described a multifaceted problem which they agreed was difficult to address. For example, many felt that stigma led to students dismissing their own symptoms of mental health difficulties as ‘just stress’, and this was seen as a key barrier in seeking support:

‘I think actually recognising it in yourself can be a difficult thing and just you can convince yourself that it is just stress and it is just normal, when in fact, it might require more extensive support ….’ Focus group.

This dismissal extended to dismissal by peers and even support providers, making students regret reaching out:

‘In first year I was a lot misunderstood by my group of friends. So they thought I was, like, bluffing or I was just stressed’ Participant 2.

‘I'm not quite sure what she [the therapist] was sort of aiming for but she kept saying that she didn't think I seemed very anxious as a person and she wasn't sure that I really had a problem with anxiety. I felt quite undermined by that’ Participant 5.

As a solution, the importance of encouraging discussion of mental health problems to ‘normalise’ the experience and allow students to feel less alone in their difficulties was mentioned. Similarly, it was felt that investing in more peer support may reduce stigma by enabling them to engage with people who understand and can help navigate the system:

‘If it's normal that sometimes people struggle and sometimes they need help from a therapist, then people are more inclined to do it’ Participant 16.

More generally, students described the shame and embarrassment associated with having a mental health problem, when students felt that they were not able to cope as others seemed to:

‘There is quite a bit of stigma around it and I think it's difficult for some students to admit that they need some support … ’ Participant 13.

However, students did highlight that the university was doing better than other universities and countries in reducing stigma, indicating that efforts had not gone unnoticed:

‘I found [the university]'s approach actually quite refreshing … [the university] are doing certainly a better job than most institutions, which to be honest could not have been hard, but they're good’ Participant 16.

### Superiority of private or external services

Although many students were grateful for the support provided at the university, some discussed reasons for seeking external support. A desire to understand the deeper causes of problems was often alluded to, something students felt was limited in university-based support options, although they acknowledged this was partly because of limitations on treatment duration:

‘Sometimes it just felt like … I could just open up about my past and everything, but then it's going to kind of wind up back to … “How can we help you cope now rather than deal with the underlying issues.” … because that takes time and that's what they didn't have’ Participant 3.

Professionalism was also mentioned by some students, who felt external support would involve a more qualified professional than was available in university services. This likely stemmed from confusion regarding available services, and which were best suited to particular mental health problems. This meant that students missed the opportunity for professional support as a result of their initial point of access being with more generic support services:

‘I think I was expecting a more legitimate psychiatry form of diagnosis and it turned out there would be no diagnosis but only sort of chatting about the problem and giving some CBT [cognitive–behavioural therapy]’ Participant 15.

As a result, students said that paying for services would mean better support with reduced waits, although some felt university services were an excellent starting point for informing further support choices:

‘That's mainly why this entire thing worked out because my parents were able to pay for that’ Participant 12.

‘It was quite helpful for me because I don't think I would have thought about seeing somebody privately otherwise’ Participant 5.

## Discussion

This study describes the experiences of university students in accessing and receiving help for mental health problems. It is intended to inform the development and adaptation of services for mental health support to students. Despite the single centre approach to this study, findings resonate with existing literature in a number of areas. Students hoped for better service integration to streamline the process of accessing support,^9^ and reduce the experience of abandonment that seemed to hinder intentions to seek further help.^7,11^ Interviewees were concerned for students who may not seek help because of stigma, uncertainty over how to initiate contact with services or feeling that the problems they experienced were not severe enough (or too severe). This corroborates previous research where students wanted more clarity on what was encompassed by the term ‘mental health difficulties’.^15^ Students also reported feeling that having experience of accessing services before arriving at university was an advantage, highlighting the importance of ensuring that students are fully aware of the routes to support and how to access services before arriving, and to be able to do so before reaching the point of crisis. However, although entering university may be a challenging time for the mental health of all students,^27^ it is not currently clear how best to engage those without previous experience of, or treatment for, a mental health disorder. Discontent with the choice of available treatment, reflected in this study, has also been raised previously,^14,15^ indicating the need for student voices to be at the heart of service design.^28,29^

Students were also not always clear about the qualifications of the treatment provider they were seeing. Although in many instances this resulted from a lack of clarity over the correct route of access to see particular mental health professionals, it also indicates that providing clear information to reassure students that all therapists, counsellors and psychiatrists are trained and competent to support their needs could be beneficial. This may also help to ensure that disparities in well-being at university are not based on differences in abilities to pay for treatment from external private providers.

The findings also raise questions of how best to respond to these emerging recommendations. A tension emerged between simplicity and complexity. Hopes for a simplified route to access, with straightforward information, were coupled with discussion of extending choices of treatment and provider. Similarly, although many students fear seeking support may result in stigmatising diagnoses,^7^ some students reported accessing services particularly to obtain clarity on a possible diagnosis in this study. These contrasting needs are challenging to resolve, but recent work^30^ has found that combining a range of on- and off-campus support services within a single ‘network’ of support, involving regular discussion between participating organisations to support the referral process, would be beneficial. This fits with students’ expressed desires to be supported through referral processes rather than feeling ‘abandoned’ to seek alternative support. Such a network could also aid the expansion of services to encompass a broader range of mental health conditions such as eating disorders; a recent UK policy document entitled ‘University Mental Health Charter’^28^ proposes that complex problems will be more efficiently addressed through combining expertise and resource, including National Health Service and other external services. This aligns with experiences noted both by students^15^ and researchers into university mental health services.^31,32^

The current study suggests that more could be done to combat feelings of invisibility. A more proactive approach to ensuring that students feel part of the university community and that there is a wider system in place to support them in their academic studies appears important. For example, some students reported that having a personal tutor who took an active role in checking on their well-being positively contributed to their experiences. Students also expressed a desire for more integrated peer support and a university-wide conversation surrounding mental health. This may also contribute to combatting stigma, an ongoing negative effect of the experience of mental health problems at university. Addressing these issues may prevent isolation and instil a sense of community,^32^ reducing feelings of invisibility and facilitating the development of a support-seeking culture, where problems are shared rather than borne alone.^33^ Peer and social support may be particularly important for international students or those from ethnic minority backgrounds.^34^

Despite these calls for change, students expressed gratitude toward those academic and mental health service staff who made a positive difference to their experiences. Although this paper focuses on how best to improve service delivery within universities, participant experiences were often contextualised with an understanding of the difficulties faced by universities, particularly those with large student bodies, in providing adequate mental health support for all in need.

### Limitations

This study has a number of limitations. First, the sample size was limited to 16 participants, and weighted heavily toward women (13 women out of a total sample size of 16 participants). Although this warrants caution in generalisability of experiences, the sample diversity in ethnicity, home or international status, and degree level should be noted. The broad consensus of opinion despite different experiences in the nature of mental health problem, the experience of care and study commitments supports the generalisability of conclusions. Furthermore, the recurrent emergence of themes suggested that data saturation was achieved, although it is possible that additional male participants may have presented additional views. Second, interviews were conducted via telephone rather than face-to-face, owing to COVID-19 restrictions. Although interviewees may have been more willing to talk freely via telephone,^35^ the possibility of yielding different findings through face-to-face interviews should be acknowledged. Finally, transcripts were not double-coded in their entirety, although themes were discussed with the research team, and validated using a proportion of the data. Furthermore, a key goal of the research was to interpret student perspectives. Within this epistemology, quantification of inter-coder reliability becomes counterintuitive.^36,37^ However, conclusions should be interpreted within the perspective of the author, a PhD student with interests in, and personal experience of, people experiencing mental health problems. The author's student status may, however, have positively contributed to limiting the potential negative impacts of power dynamics on discussions, through encouraging a more open and frank discussion with students compared with that which might have been achieved with a more senior staff member. The conduct of a confirmatory focus group with participants to discuss interpretations acted as a further means of triangulation, alongside discussion with co-authors, to check potential biases and reasoning.^38^

In conclusion, this study further contributes to the existing evidence indicating the need for simple and clear access to mental health support for students, and an approach to treatment that involves collaboration across university and external mental health services to ensure support is available to all who need it. Themes suggest that current experiences can contribute to feelings of isolation and abandonment, and additional efforts to establish peer-support networks may provide a beneficial platform to normalise mental health problems. In line with recent recommendations, student voices must be an integral part of service design.

## Supplementary material

For supplementary material accompanying this paper visit http://dx.doi.org/10.1192/bjo.2021.947. click here to view supplementary material
