# Supplementary material for: ‘Five hours to sort out your life’: qualitative study of the experiences of university students who access mental health support
Source: BJPsych Open. 2021 Jun 24;7(4):e118. doi: 10.1192/bjo.2021.947 (PMC8269927; doi:10.1192/bjo.2021.947)
Supplement: Supplementary file 1 [file S2056472421009479sup001.docx]

# Appendix 1: Interview schedule and adaptation based on student experience

**Topic Guide**

*[Before recorder]*

*-introduce self, re-explain what the study is about, consent, confidentiality, no right or wrong answers, right to withdraw at any time*

*[turn on recorder]*

1. **EXPERIENCE OF CARE**
   1. To give our discussion a bit of context, could you tell me a bit about your general experiences of mental health problems and treatment?
   2. ~~You have accessed some of the student mental health services at UCL, cAnd~~ could you tell me about your experience of accessing the student mental health and wellbeing services at UCL?

[Questions to ask if not covered]

- - 1. *Specific services accessed*
    2. *choices*
    3. *Waiting times*
    4. *Difficult or easy to access support?*
       1. *Did the services seem approachable?*
  1. Can we discuss how you think it helped (or didn’t help) you?
     1. *relevance*
     2. *sufficiency*
     3. *time provided for advice and treatment?*
     4. *Was undertaking treatment difficult during your studies?*
        1. *E.g. side effects, emotional toll of therapy*
  2. Did you access the student mental health services because of a pre-existing mental health condition or because of development/exacerbation of symptoms while at university?
     1. *What made you seek help?*
     2. *how long until you decided to seek help?*
     3. *Had you accessed mental health services prior to starting university?*
     4. *If so, what support had you previously accessed, and how did you go about obtaining this support?*

1. **KNOWLEDGE OF CURRENTLY AVAILABLE HELP AND HOW TO ACCESS IT**

We are now going to focus on the information provided to students at university…

- 1. What potential sources of support for mental health ~~problems~~ were you aware of prior to starting at UCL?
     1. *Had you searched on the UCL website?*
     2. *Other forms of own research? Was information easy to find?*
     3. *Were you sent information? Were your parents?*
     4. *How did you know about these sources (if none of the above)*
  2. Was mental health services information provided when you started at UCL?
     1. *Fresher’s week/induction week?*
     2. *Information in halls?*
     3. *Emails?*
     4. *Tutor/department?*
     5. *Transition mentor?*
  3. IF INFORMATION GIVEN What support were you told about?
     1. *E.g. campus based? Community and NHS support? GP? Academic staff to speak to?*
     2. *Did you find any of this information useful?*
  4. IF WERE NOT GIVEN INFORMATION: what information, in hindsight do you think would have been most useful to receive?
     1. *When would be most useful to be told about mental health services?*
     2. *~~What would be the best way of getting information to students~~What would have been the best way of communicating this information to you?*
     3. *What mental health services would be most useful to be told about?*
        1. *Campus based wellbeing advice*
        2. *NHS services?*
        3. *Student counselling services*
  5. In what ways do you think the information communicated to you via UCL is effective? ~~How do you think current information provision at UCL affects the process of accessing treatment?~~
     1. *~~Does~~ Do you think ~~it make it easier/harder?~~ it impacts access to treatment?*
     2. *Too much/too little?*
     3. *How did the information affect your expectations of the process and subsequent experience?*

1. **BARRIERS**

I would now like to talk about anything which may prevent you (or others) from getting the help they need…

- 1. Did you experience any obstacles whilst attempting to access support?~~barriers to receiving support?~~
     1. *Anything that caused~~meant~~ you to stop~~ped~~ receiving support sooner than you would have liked?*
     2. *Anything that made getting the treatment or support you needed more challenging?*
  2. What are your thoughts on mental health stigma?
     1. *Impact on seeking support?*
     2. *Can you think of anything the university could do to reduce this?*

1. **EXPECTATION**

Going back to your experience of using the services at UCL, we are now going to think about your expectations and whether they were met…

- 1. How did your experience of the support you received compare to your expectations?
     1. *Waiting times?*
     2. *Range of treatment options?*
     3. *Availability and flexibility of services (e.g. out of hours care)?*
     4. *Treatment provider?*
     5. *Efficacy of treatment?*

1. **RECOMMENDATIONS**

And lastly I would like to hear your views on improving the services…

- 1. Do you have any suggestions for further ways ~~How do you think~~ UCL could improve ~~the way in which they provide~~ mental health support and treatment?
     1. *What would encourage more students to attend/limit the people who stop attending?*
     2. *Is there anything you would have liked to have access to but didn’t?*

-Thank you

-anything else you would like to add?

-Any questions for me?

*[switch off recorder]*

-thank participant for their time, arrange payment, any other questions now recorder switched off? Explain process for withdrawing data.

# Appendix 2: Subthemes and example supporting quotes for personalisation and informed choice

| Subtheme | Description | Example supporting quotes |
| --- | --- | --- |
| Services for all disorders and all levels of severity | It was frequently highlighted that current support options excluded certain presentations of mental health problems, which it was felt discouraged students from speaking out about the difficulties they were facing, because they were encouraged to instead seek support via external means. This was further highlighted during discussion in the focus group | *“I know they’re trained to help and to send me to the right place so that I can get help but I don’t know if it’s laziness or just when you’re anxious you really don’t want to go through a lot of, you know, admin things and applying somewhere else. I really just wanted to find a sort of simple way of finding support.”[P10]*  *“they were just like, if you have any official mental health disorders like eating disorders or specific like OCDs or phobias or something, they won’t be able to do anything for you” [FG]* |
|  | It was emphasised that eating disorders are sufficiently prevalent that efforts to provide appropriate support would be worthwhile. Focus group discussions confirmed this. | *“I find that it revolves a lot around anxiety, depression and that but they don’t really talk about eating disorders, for example, which are really present in [the city] and our age, in your young 20s and I feel like that’s really something that’s common and that’s really not spoken about. And maybe that’s something that could be, that personally I wish I had – with you know, in the myriad of fliers that I got in my first year, I wish I’d gotten one on that too.” [P13]*  *“I didn’t see that UCL had that much of a counselling service for people with eating disorders specifically, so I kind of know why they couldn’t help me.” [P15]*  *“so maybe even having just like a triage session to know whether someone needs something more intensive. And having a fuller range of services, because eating disorders are a very prevalent thing, especially at universities, so I feel like the option at least needs to be there.” [FG]* |
|  | Students were not always certain that current options were adequate for severe presentations of mental illness, and students with more complex issues were told to seek help elsewhere | *“I think if there was a crisis service that would be amazing” [P10]*  *“I don’t really know personally this sort of stuff, but it seemed more like a chat rather than, like, actually addressing the problem. I don’t know. It might be that sometimes people just need to talk with someone and I would say that it’s good, but when – I don’t know. I wouldn’t really go there and tell them that I am suicidal and I want to hurt myself because it just doesn’t seem the place.” [P2]*  *“I saw someone from uni late in March and I was told that for me the best option would be to go to private therapy if that was an option, just because uni offers kind of more short-term support, if there’s kind of like quite a defined issue you need help with, like a specific thing.” [P12]* |
|  | It was also highlighted that students wanted a service able to offer mental health support to whoever may need it, regardless of severity, to reduce worries by some students that they were not experiencing problems severe enough to seek help | *“maybe being clear about what they are there for and what sort of issues they’re there to address, really. Definitely that would, I think, be a helpful thing. Sort of make people feel like that services are there for them rather than someone else.” [P4]*  *“I think it probably goes back to some of the things we’ve talked about before, about just you know, kind of making it clear that there are sort of signposting kind of services available as the first port of call and that you don’t have to wait ‘til, you know, ‘til you’re literally kind of crippled with anxiety or paralysed with depression until you ask for help.” [P5]*  *“And then also like, yeah, because not everyone will identify with like being mentally ill, like they might just, they might not um, but they might still really benefit from that mental health support. So I think, yeah, there’s a lot of like, people might read Psychological and Counselling Services, sort of like something like that and think “Oh that doesn’t apply to me”, when really it really would.” [P6]* |
|  | Some students suggested that advertisements/information for services should describe the difficulties experienced rather than mental health problems (diagnoses), to prevent students who are unsure if they were experiencing difficulties severe enough from not seeking help | *“Maybe more like descriptive stuff like “Are you struggling with X, Y, Z?” Like more kind of scenario things that people can identify with rather than just like “Are you struggling with your mental health?” So if it’s like “Do you feel overwhelmed? Like have you got all these deadlines? Like you haven’t done your washing and like haven’t eaten and like no one to talk to, like maybe you would benefit from this kind of stuff”.” [P6]*  *“I think sort of breaking down the idea that the Psychological Services are only really there for students who have some kind of diagnosed psychological issue that, you know, is – I think that there’s this sort of really high bar for accessing those services, you know, that you have to get, wait until things are really bad to get support.” [P5]*  *“ just the message that sort of it can be tricky to sort of see whether, what are they going through actually kind of validates professional help but just a message of like, it is of course subjective, but if you’re feeling unsure and if you’re struggling with something, it’s probably a good idea, like it doesn’t need to be like the full blown mental health crisis or everything is falling apart. If there’s something that’s really bothering you, it can be a good idea to just sort of check and see.” [P12]* |
|  | One student also suggested that personalising treatment plans to different severities of symptoms could be one alternative to the ‘blanket’ 6 session offering currently available. This was supported during the focus group, with a recognition of the need to have the option to step up support for students who need additional help made clear. | ***“****So I don’t know whether like UCL can provide like the full Psychological Services that like a NHS provider would provide. It would be good to have variations in the duration of sessions.” [P8]*  *“Yeah, I think it seems like a very one size fits all approach and yeah, I think it could potentially be very harmful in the sense that, I guess a lot of people might be experiencing mental health issues at university for the first time. And if they’re getting insufficient support, then I guess that leaves you incredibly helpless, because you’ve had the university support and you get to the end of that and potentially still feel like nothing has really been addressed. And it’s a bit like, ‘where do you go from there?’ kind of thing.”[FG****]*** |
| Taking preferences into account | Students stated that they would have appreciated some level of choice in the support provided. For example, students frequently alluded to the idea that they felt they had to be grateful for what they were given, and accept it without request for alteration or complaint. This was further agreed on during focus group discussions. | *“And then I think once you’ve got that information you can make more of an informed choice rather than – because I think at that point I was just, I was kind of pleased to be getting anything.” [P5]*  *“I think where like I’ve had so much interaction with mental health services and I’m very aware of like the lack of funding and issues that face the people working in these services, like my expectations were quite low.” [P6]*  *“for me, it was like a pressure to feel like I should be grateful for it. Like, given the state of the NHS, the underfunding of mental health services and a long waiting list with those, then just the fact that my university was offering something. I felt like, even though it was only six x 15-minute sessions, which is like five hours to sort out your life, I still felt like I had to be grateful that I’d been seen” [FG]* |
|  | Students also suggested that being able to choose the treatment they received would have made their experiences more positive. | *“I think one big thing for me was because after – because when I was in treatment at home, that was group therapy based and I sort of had a very good experience of that. And when I came back, I initially would have liked to join a sort of self-help group or like something group based. And I couldn’t really find anything, like my GP didn’t know, Psychological Services couldn’t really point me anywhere” [P12]*  *“I think I was lucky to get a family relationship therapist, because that was related to the problems I was having. But imagine if I didn’t have that then I’d just feel like, where’s my choice? Couldn’t I have chosen a therapist that specialises in something that is applicable to me?” [P3]*  *“I kind of asked to see a CBT, asked to have a counselling service as well, but the admin say that I, that whether I get referred to have CBT is up to my psychiatrist.” [P14]* |
|  | One student reflected on a recent more positive experience where this was the case. | *“I definitely felt the second time round that the kind of, there was flexibility and that there was – that it was kind of, the support I received was tailored for me rather than just a kind of a broad brushstroke, “Well here’s what we have”, as a, you know, “Here’s your single option, take it or leave it”, kind of thing.”[P5]* |
|  | Though it was appreciated that in a large university, personalisation for all may be difficult to achieve. | *“I think that the main problem with UCL is that they’re trying to have kind of a general approach to everyone but not everyone reacts the same way to this general approach. And it’s wonderful that they have this but it’s not – I mean they can’t make it different, they can’t tailor it to every single individual but every individual needs a different way” [P13]* |
|  | Similarly, some students felt that their experience may have improved with choice surrounding treatment provider. This notion arose due to expressions that students did not get along with, or “click” with the therapist they were given, either due to negative interactions or feeling that their therapist could not relate to them due to differences, for example, in ethnicity. | *“I think it really depends on the person. And in the wellbeing services I had amazing experiences, most of them were really great experiences but some of them were really bad. And so I think it really depends on the person and what their specialisation is. Because some will understand more easily autistic traits and others won’t and yeah, I think it’s important to sort of really, to be with the right person.” [P10]*  *“I think I also, I found it, I struggled to get on well with this particular counsellor, probably, and probably it wasn’t helped by the fact that she was quite unreliable in terms of turning up for sessions but she – I’m not quite sure what she was sort of aiming for but she kept saying that she didn’t think I seemed very anxious as a person and she wasn’t sure that I really had a problem with anxiety.” [P5]*  *“So it almost feels like it’s hard to speak about things that the person doesn’t necessarily understand or relate with. So maybe kind of going into the situation wanting to talk about those kind of issues and not having a person with like that diversity of experience might put somebody off” [P8]* |
|  | However, this was not universal, and some reported very positive experiences with their counsellor. | *He was great, really nice, very insightful, so I had a really great experience with my counsellor actually” [P15]*  *“I was really happy with the therapist that they matched me to or that I got to see…And when I reapplied actually even,… I didn’t hear anything back from them so I kind of went and got, sorted my own therapy. But when they did get back to me, they offered me appointments with the therapist that I saw before which I thought was really good because I got on with her really well and I thought that was like, that was something really good.” [P9]* |
|  | Finally, students appeared to have had different experiences in flexibility for appointments | *“Well I think one thing that I remember finding quite difficult when I was accessing it, was the clashing with my timetable.” [P8]*  *“it surprised me that they were willing to be open very early, before the uni lectures start.” [P7]* |

# Appendix 3: Subthemes and example supporting quotations for simplifying the process

| Subtheme | Description | Example supporting quotes |
| --- | --- | --- |
| Importance of collaboration across services and staff | Almost all students mentioned links between support staff and academic staff, however different views were given regarding how connected these two strands of university staff should be. Almost half of the interviewees acknowledged that some students would be put off by the knowledge that academic staff would be informed about any mental health problems they were experiencing, due to a concern that tutor perceptions, or even grades would be influenced, suggesting that confidentiality is an important aspect of support at university. | “ between me and the department there are some power relations. Like, they mark my work, they determine whether they admit me as their, like, future student if I want to study further and if I want to apply to the same department”[P1]  “I think people are always scared of this issues impacting on their grades or their academics, and just make it really clear that even if you go and reach out for help, it won’t impact you at all, or it will impact you just positively – not your grades or anything.” [P2]  “I think it should with the permission of the individual student. So if they were saying like “OK, I think like these practical things that your department could do in order to support you would be useful, like do we have your permission to contact them or like can we support you in asking them for these reasonable adjustments?” Like I know that the Disability Service are quite good at this like with the creation of Summary of Reasonable Adjustments like the SORA stuff.”[P6] |
|  | However the majority of students subscribed to the idea that having a member of academic staff or personal tutor who understands mental health problems can make academic stressors less prominent issues which would be a beneficial way of providing a clearer route to support. It was also felt that feeling that academic staff don’t care impacted negatively on the university experience. Focus group discussions supported this view. | “ And so it would be to have personal tutors who are more accessible, so personal tutors who are willing to take on the role of being a personal tutor and not just an academic advisor that we never see or come into contact with. I think that would be really good.” [P13]  “I’ve spoken to friends who have gone to their personal tutors, like different personal tutors, and had like such a bad time with it just because their personal tutor was clueless and kind of was like “Oh I don’t really know what to do”. And then that’s really disheartening and then you end up feeling like, they felt like “Oh there’s no hope”, from that poor interaction.” [P6]  “there’s a few who are great advocates for like all the help that’s available and they’re really clued up on it. But I found in general my professors, like academic staff, were sort of a bit more blasé about it. Like they sort of know it’s there and they say “Oh have you sort of spoken to someone?” And if you say “Oh yeah”, they’re like “OK, good, good” and then they sort of move on.” [P9] |
|  | Those students who had experienced a personal tutor who understood their issues, spoke positively about it | “And yeah, that was again quite fortunate that my tutor was really nice and approachable and understanding. Yeah. I think that was sort of a fortunate aspect of it in that I can think of – I don’t know, I think it was quite nice. He's just done his PHD so he’s not that far away in age and he’s sort of just quite kind of unassuming and I think having him as a tutor was quite good in that it did sort of feel like the things I was struggling with I would be able to talk to him.” [P4]  “He knows everything about my disorder and he’s helped me to defer exams or make adjustments. He has helped me to kind of manage my time better or when I was not going to lecture, he knew all about that.” [P2]  “my personal tutor is exceptional, I got very lucky with her, she’s also like a – she’s a qualified doctor, that she’s, yeah, she’s practicing medicine, so she’s very clued up on mental health.” [P6] |
|  | It was frequently suggested that as a minimum, academic staff should be trained to help point those in need of help in the right direction to facilitate access to support | “ it would have been helpful if maybe my tutor had also pointed me to sort of the resources available and just kind of in case you need further academic help or maybe a bit more support with just general stuff.” [P12]  “But I think the academic staff need to be more aware of what support is available. So like my supervisor, it would have been really helpful if he could have also sort of pointed out some of these services to me rather than what really happened which was me kind of making him retroactively aware that I was seeking support from X, Y and Z.” [P5]  "I think there is like a procedure that personal tutors are meant to take but maybe they need more training so that they kind of know what to like look out for and know what to say or where to signpost to.” [P6] |
|  | Similarly, the need for collaboration between different support services was discussed by many students who reported being confused about who they had seen, who they should have seen, and who they might see next. It was made clear that a first port of call when experiencing mental health problems could improve the experience of accessing support as it would take the guesswork out of deciding who to see. This was also agreed on in the focus group | “I think it would be even stronger for UCL to have a very good team that help students to find help, rather than providing help, if that makes sense….I think it should be actually left at that, it should be left very simple way to say, like just one place that people can go if they need help, just one place saying “I need help”” [P11]  “what is a problem, one issue I have with the two service is I wasn’t clear the difference. I wasn’t clear what are the differences between these two service, something like that, and what are the limits, what are the remits that they have, if I can ask for help, does that make sense?” [P14]  “I guess maybe it would be more helpful if there was kind of a, more of a kind of a tiered entry I guess, kind of, you know the first, your first sort of contact with the services is actually not necessarily aiming to be sort of therapy at all, but just exploring what your problems are and like what would be helpful….I think if there was kind of a, I guess kind of a signposting service as it were, as like an initial point of contact, that might also help remove that barrier of like well are things bad enough yet or, you know, should I be trying to help myself more in some way that I haven’t done yet or? Because it could also point you towards those things, you know, it doesn’t have to be an all or nothing answer… I thought that those kind of mental health support services would all be done through SPS, like I hadn’t even thought that that would be done through Disability Services. I think it would be amazing if those services could talk to each other more because there’s some really good support services at UCL but if you don’t know that they’re there, than you can’t use them.” [P5]  “I feel like if I was approaching it for the first time, I wouldn’t necessarily know what was going to be the right thing for me. And I feel maybe a more unified system in which there are more options, but equally, that that service would be better at maybe having a bit of assessment and then directing you towards a specific part of the service that they think is right for you. Maybe that might be important, because I don’t think, necessarily, you know, when you’re first approaching this kind of thing, like what is going to be helpful and what isn’t.” [FG] |
|  | A referral was also considered as rejection by some, who highlighted the importance of preventing students getting referred for more intensive or external services from feeling abandoned, for example through providing support and follow up during the process. The problems associated with being referred on, such as lack of rapport with any therapist, were elaborated on during the focus group. | “I found it very distressing because I don’t like changes, it’s one of my autistic traits. And all these changes and all this, I don’t know, being sent somewhere else, where I was, because I was, I have a lot of anxiety and I just, you know, needed to speak to someone.” [P10]  “Well like some kind of like buddy system, of like “Do you know what, like you need to go to your GP, let me come with you”. Or like “Here’s like a – let me plan out with you like what you need to say specifically to your GP in order to access help”, because that in itself is really hard.” [P6]  “Yeah, and especially people dealing with a variety of issues, and if you’re dealing with something very serious, you kind of want to be with somebody that you trust. And I think that might go if you’re being bounced around from person to person.” [FG] |
| Uncertainty | Difficulty navigating the multiple services on offer, and the sense of uncertainty this could instil, was elaborated on by most students. Over half suggested that in order to understand the system and access the support you need, you needed to have had experience of similar systems and services prior to coming to university, suggesting that the current framework may exclude students experiencing difficulties for the first time . | “I’ve done this a lot of time, because I don’t know – even the process of extenuating circumstance, a lot of people, they don’t know even how it works.” [P2]  “I think what made it good is that I knew I had a very clear idea of what I was struggling with and what I needed support with and I was able to communicate that to them… because of the help I had before, like when I lived in care, like I had like 24 hour staff, like literally taught me skills of like “This is how you send an email to explain like what is happening and what you need. Like this is how you identify like the problems you are having and you communicate like what would be helpful” Whereas I think like people that haven’t had that support, like if you’re experiencing a mental health problem, like it feels impossible to describe what you’re experiencing, let alone like ask for what you need” [P6]  “Yeah. So I, pretty much as soon as I came to UCL, I got in touch with Student Wellbeing because I already kind of foresaw that I would probably have some difficulties at some point, so I just sort of wanted to like get in touch with them as soon as possible…Because I found, you know, I assumed like it would be similar to the NHS in that like the more proactive you are about like, um, contacting people and making yourself known and stuff like that, the more like easier it is to get help when you need it.” [P9] |
|  | Students with less previous experiences of mental illness described the uncertainty they felt about what they should expect from attending mental health support services. | “And I think I didn’t have a very clear idea of what they could actually offer to me in terms of sort of therapy or support or counselling or whatever.” [P12]  “Well, yeah, because it’s like a random therapist kind of assigned to you, and even after the first assessment it’s not done by the therapist that is going to be with you for the six weeks. It’s done by someone else, so you just really don’t know and you kind of just have to go with it. So yeah, I guess I was a bit nervous and sometimes you just wonder, would they get me? Would they get my culture? Would they just be a big judgemental, kind of thing?” [P3] |
| The need for clear, simple information on accessing support | Almost all students talked about the overwhelming array of information available, which was seen as lacking in clarity or a clear directive on where, when or how to go about establishing contact with the support services. | “In the enrolment day they all distributed a booklet to each student about the Mental Health Services or Wellbeing Services. And it’s like – I don’t know. At that time I flicked through it, I felt like it’s a lot of information, it’s too much – that I don’t know what to choose and which one best suited me.” [P1]  “I think, so in Freshers Week, I think there should be, I don’t know, some kind of information that sort of sums up everything but on the same page so that you can really compare the different services, whether it’s for disability or for coursework or for mental health, there’s sort of three options and I’m not even sure because I’m quite confused.” [P10]  “Like if you are in a stage that you need help, it’s really time consuming and overwhelm – it feels overwhelming too. I know there are many information out there but just looking for these things is just, and sometimes you know having ten options, it’s worse than having just one.” [P11]  “I think just unloading all the information in the first few weeks might be a bit too much, but I think offering – starting with a general guidance and then going into more specific discussions of support I think would make a difference, because then people who are looking, generally looking, will go to the sources of information that they’re given.” [P7] |
|  | Many students wanted the information available to make the range of options available at the university and externally clear as they were not aware of some of the treatment choices available to them. | “I think it would be helpful, there could be at least like a general breakdown of like “Oh you can sort of, you can go through the NHS, you can go through charities, you can go privately”, just sort of, even like a very basic table and maybe at least a few points of contact you could seek out.” [P12]  “But when I was waiting to meet my psychiatrist at the waiting room, I was told by other students who were waiting as well, that they are waiting to see not psychiatrist but psychologist and they have to get assessed to other counselling service but they wasn’t aware that they can meet psychiatrist as well….So I wasn’t aware that they have a different service for me and they weren’t aware that I’ve got a different service from them as well, which I think is down to the problem that we didn’t really understand what could be the option for us.”[P14]  “because I didn’t realise how easy it was to access things like CBT and like the fact that you can even self-refer. And I think if I had had access to that information a lot earlier in my course, like things wouldn’t have been as difficult as they were.” [P8] |
|  | Some students felt that information they did receive was not always accurate or complete- for example some students expressed shock at the waiting times and limited sessions provided after contacting services. It was also acknowledged that prior warning of waiting times may prevent students seeking help | I don’t remember it being clear to me that it would only be six sessions though.” [P6]  “Yes. I think for me it would have been helpful, that’s just a small thing about kind of my specific route. If they’d given me kind of, even just a vague estimate of the waiting time, so that I would have known that kind of help was still coming.” [P12]  “I was quite surprised at the end of the session, I was expecting well “I’ll see you next week”, and actually it was “Well you can go there, there and there”.” [P10] |
|  | Despite this, some students did feel that once you knew it was there, information available online was helpful | “Yeah, yeah. I think the most important part is that people know that it’s there, that the information is there so that it can be found if they’re looking for it.” [P16]  “I just googled it” [P9]  “In terms of, like, where to go and how to access stuff, the instructions online are pretty simple” [P3] |

# Appendix 4. Subthemes and example supporting quotations for feeling abandoned, ignored, or invisible

| Subtheme | Description | Example supporting quotes |
| --- | --- | --- |
| Faceless and lost in the crowd | The sense of loneliness and invisibility felt due to being one of many students at a large university was clearly illustrated in students descriptions of their experiences. | *“Yes. I think just to me kind of, in my first year university didn’t always feel like a very sociable and caring place.” [P12]*  *“it feels extremely anonymous to be filling out a form online about what’s going on for you and how, it’s really scary..” [P13]*  *“or try and make university more personal, because I feel like you can get lost in the crowd of, like, 300, 400 people on your course, and you can just feel like a number sometimes”[P3]*  *“I think one of the things that was really sort of highlighted to me this year was just how sort of easy it is for things to kind of – I don’t know, for people to be struggling with things, but for it to very much slip under the radar just because of the way uni is, really.” [P4]* |
|  | And this sense was exacerbated by the extensive online forms required in order to see and speak to someone who could listen. Focus group discussions agreed that this administrative barrier was present across a number of services. | “Well I think that what’s helpful is that having, so it’s important to have physical contact and not just, you know, fill out forms online and not know where to go in the building and everything…. it seems like there’s so much – bureaucracy maybe isn’t the word, but so many steps to getting somewhere, to getting actual concrete help with your problem.” [P13]  *“and I don’t even think it’s just an issue with the psychology services. I think it’s an issue throughout postural support where it feels like there’s a lot of hurdles in order to actually discuss problems that you’re having.” [FG]* |
|  | As a result, students emphasised the importance of an environment which felt welcoming as an important contribution to positive experiences. Focus group discussions elaborated on this, describing welcoming atmospheres as contributing to feeling less invisible and more like there are people who care. | *“Yeah. OK, like, at first I really didn’t know what to expect, but while I was waiting the other warm advisors gave me the impression, the expectation that I will also be treated in a very gentle way. But in the end the reality is like, I waited for a long time and, like, in the end when I came in her room, she just wanted me to leave. It causes a bit of disappointment.” [P1]*  *“I think they need some sort of like – like especially for freshers – they need some sort of team or something, like a representation of people that you could go to with events and just friendly, open people. So that it just feels like somewhere you can go to and not be judged and everything, and feel kind of safe. can feel safe. Whereas I think now, and especially when I first came to uni, it was a very cold, hard kind of thing, like ‘oh, if you have problems, just go here’. Like it just felt like a service, whereas I feel like it should feel more like a family or a community that’s willing to help and listen and understand stuff.”[FG]* |
| Feeling let down | Some students described feeling let down by the services. | *“I started going to therapy and doing it privately because I just, from what had happened with the second year experience, with trying to reach out to UCL and not getting a response back, I just didn’t feel like I would get the support that I needed and so, yeah, that’s kind of what it’s been.” [P13]* |
| Abandoned | This feeling sometimes arose when being informed of long waiting times, or when trying to seek help at crisis point and resulted in feelings of abandonment at their lowest point. | *“That [the wait] was very difficult just because I didn’t have any sense of like how long it might take…. I thought that wasn’t going to happen, I’d almost forgotten about it, just because it had been so long.” [P12]*  *“at the moment as you are, let’s say severely depressed or you’re really struggling, you just want help at that exact moment. You don’t know what in that two month period, what’s going to happen and you don’t really – after two months you’re not in the same mental state anymore, so it just made me a little bit disappointed and a little bit untrusting towards the whole system.”[P15]*  *“When I first applied I didn’t get an email, like a message or anything until a couple of weeks or possibly months later, and even then they were just like “you’re on a very long waiting list – you’ll have to wait for a very long time.” And then once they did email me it was just, like, this is months from when I first emailed. And yeah.” [P3]*  *“I think that time between, you know, filling out the application and actually first getting seen, starting the therapy, like luckily I was able to kind of cope through that time but I’m sure a lot of other people wouldn’t have been able to if they had similar circumstances.”[P8]* |
|  | Though others felt that waiting times were to be expected given the large scale of support required for a large university and were not surprised, or did not experience too much distress when waiting. | *“So I mean, the waiting time was obviously something that could be improved on, right? I’m not sure where the problem is – maybe it’s facilities, maybe it’s staff, maybe it’s budget. So might not be a thing that can be fixed short-term but that would be certainly a point that could be improved.” [P16]*  *“I found it [waiting time] great. I saw someone very quickly.” [P10]* |
|  | Limits in the number of sessions provided, seen as necessary to prevent even longer waiting lists, were also a source of feelings of abandonment as students felt they were left to deal with their problems alone. This was agreed in the focus group. | *“You have your six weeks, it’s over, find something else, go back to your GP, check out these websites, et cetera, et cetera.” [P3]*  *“the therapist they had was really good. She really tuned in to my problems and she listened really well and she gave some really good pointers and everything, but it was very short-term. Like, the timing as just insufficient, and you could feel that as you were going into the therapy, because they did say it is six weeks.” [P3]*  *“So I remember like when the therapist who was really – I thought she was really brilliant, when she said like “Oh and next week is our last session”, I remember being like quite shocked and then I thought like oh actually like that’s quite representative of like the mental health service in the UK, but um, sadly.” [P6]*  *“then I guess that leaves you incredibly helpless, because you’ve had the university support and you get to the end of that and potentially still feel like nothing has really been addressed. And it’s a bit like, ‘where do you go from there?’” [FG]*  *“no one’s going to open up about that [an eating disorder] on the first session. They might say it like – I don’t know – on the fifth or sixth session, and by then, your six sessions is up.” [FG]* |

# Appendix 5: Subthemes and example supporting quotations for stigma

| Subtheme | Description | Example supporting quotes |
| --- | --- | --- |
| Dismissing symptoms as stress | It was frequently hinted that a major problem in students was their own self stigma, where they dismiss their difficulties as stress, a common aspect of university life which they should be prepared for and which they should be able to cope with | “it’s just – another thing that I’m kind of blaming myself a bit for not, for having mental health is because I can’t deal with my stress and relaxation and my lifestyle and juggling with my routine, something like that.”[P14]  “I think lots of universities have this issue of kind of, it’s almost expected that you’ll struggle at some point with anxiety around your course or whatever and sometimes sort of disassociating those things is quite hard… And I think there’s definitely this feeling that, yeah, that kind of, that sort of work related stress is really, yeah, kind of expected and not something that you ought to seek help for. “[P5]  “it’s quite hard to self-recognise ‘at what point is this reactionary stress and an appropriate level of stress?’ And then at what point does it dip into ‘I need actual help’?” [FG] |
|  | It was also mentioned by some that this dismissal came from others, including peers and even therapeutic providers, which contributed to fears they were over-reacting or just not able to cope with university. | “I know that some people are suffering from the stigma of, I don’t know, being depressed and then someone telling them “Oh it’s fine, just go out and hang out with your friends”, whereas depression is an illness.” [P10]  “some people think mental health problem is kind of, it’s kind of happened to people who can’t deal with their emotion or they can’t deal with their relaxation” [P14]  “I’m not quite sure what she [the therapist] was sort of aiming for but she kept saying that she didn’t think I seemed very anxious as a person and she wasn’t sure that I really had a problem with anxiety. I felt quite undermined by that I think because it really was a problem for me and I had intermitted because of it and I was feeling very anxious.” [P5] |
| Raising awareness and normalising mental health problems | In discussing solutions for stigma, most students felt that more could be done to raise awareness of the difficulties faced by students with mental health problems, broadening conceptualisations of what constitutes “enough” to warrant seeking support. It was felt that ‘normalising’ the experiences would contribute towards increased numbers of students seeking help at the university. | “ The ideal thing would be to sort of erase this kind of difference there is between mental and physical health.” [P10]  “I’m not sure exactly what they could do but definitely you talking about it and maybe associating it with more general health.”[P11]  “like having a workshop in groups in your department where – like in the same way they do like the, well they try to do the compulsory consent, like sexual consent workshops, like having stuff like that where it’s like “OK, what does poor mental health look like? What’s available to you?” “ [P6]  “maybe even just having more regular information going out, just generally about mental health or like encouraging, I don’t know, like if you’re going to do it in a seminar group type of thing, but just regular check-ins so that people kind of normalise it a little bit more.”[P8] |
|  | The majority of students also spoke of the need for more open and honest discussion among fellow students regarding mental health problems, in order to reduce the stigma surrounding it. The idea that realising you are ‘not the only one’ would be helpful was often mentioned. This was added to in the focus group discussion, where students felt that many students put on a brave face, which leads to others not wanting to seek help because they feel they are the only one with difficulties. | “I suppose it’s hard to know as one person whether what you’re experiencing is normal or not…you can share your experiences with others and that you’re probably having at least some similar experiences without really knowing it.”[P12]  “And also amongst friends, so we can all talk about how nervous we are for exams or for whatever it is, but then really talking about the deeper personal issues that wouldn’t be appropriate in that context or with new friends in your first year or something like that, I think it’s difficult to kind of overcome like that sense of being alone in it or shame maybe a little bit too.” [P13]  “And students as well, I feel like it’s a large group of people are displaying this mentality that they never get stressed and they’re always on top of things and everything. Other people would want to do the same as well, and then, before you know it, it’s like half the year. Just like everyone’s on it and everyone manages to do everything and look well-rested, and ace all the tests and have a good social life and everything.” [FG] |
|  | Along a similar line, half of the students felt that developing this into an official peer support scheme could go some way to reducing stigma, through sharing experiences, getting help from people who understand and helping to navigate the system. | “have students who talk about the steps that they took and how it helped them and how – just to make it clear for students, for other students who want to take on the act of looking for support in UCL. “ [P13]  “seeing people who are successful but still struggling with these things, having the opportunity to talk to them and talk about their experiences, I would have thought that very helpful in the past” .[P16]  “Well like some kind of like buddy system, of like “Do you know what, like you need to go to your GP, let me come with you”. Or like “Here’s like a – let me plan out with you like what you need to say specifically to your GP in order to access help”, because that in itself is really hard.”[P6]  “Yeah, because in my course they did have a kind of medic mums and dads thing. So like a person from the year above was there to kind of support you and I think just generally having somebody to, you know, relate to and who has been through the experiences really does help. So maybe having that as like a more official wider scheme could help as well.”[P8] |
| Shame or embarrassment | More generally, students spoke of the shame and embarrassment ingrained in the experience of mental health problems at university as a result of mental health stigma; a notion validated during the focus group discussion. | “So there was a bit of shame, I would say, and I think it does prevent a lot of people to access support. I was in Mental Health Society, like part of the committee last year, which is a new society at UCL, and we – well, this society didn’t go very well because for instance I thought because it’s a new society, but also because people don’t want to be part of a society that’s called UCL Mental Health Society, because they just don’t want to be seen as having a mental health issue.  And so we experienced a lot of people acting really weird, especially at the freshers’ fair. Like, they were even scared of approaching us at the freshers’ fair, so yeah, I think that might be a big setback.” [P2]  “it almost feels like I kind of have to either mask the problems that I’m dealing with or just not talk about them because it would do me a lot of damage.” [P8]  “that idea of shame and guilt and wanting to kind of hide away the fact that you’re using those services.” [FG] |
|  | One student spoke of the particular stigma surrounding medical students who they felt others believed should always be able to cope. | “I mean, for me, the most stigmatising thing is – because I’m a medical student, that medical students should be, like, perfect in every aspect and shouldn’t really have mental health problems, otherwise why are they going into such a pressurised job? Why can’t they just work in something else, if you’re that stressed or if you’re that anxious or if you’re that depressed or anything? So it kind of just, like, puts people off admitting that they have mental health problems.” [P3] |
|  | A small number of students felt that stigma may become a particular barrier to seeking support due to issues surrounding privacy when attending some appointments. One student also felt that confidentiality could be highlighted more to reassure students. This was elaborated on during the focus group, where a student described the lack of private space to speak to people on the phone, something students may not have at home or in halls. However, also during the focus group, one student disagreed, indicating that not all students had difficulties surrounding privacy. | “Even the fact I think that it is inside the student centre, that is a bit weird, because then people see you go in” [P2]  “Yeah, yeah. I think it might be sort of a little bit more comfortable to have somewhere a bit more private. Because basically everyone can see you walking in there, really. Yeah.” [P4]  “because London is so busy, and especially UCL, the buildings are quite busy and if, say, you wanted to talk to someone on the phone, because you’re having like a horrible day or you’re having like a crisis or something, there’s literally nowhere to go. Like there will be people anywhere. Like there’s this one corridor that’s usually quite quiet, but then people still walk through it in one of the buildings. So I feel like there should be like a space for people to go if they need to talk to someone, like not necessary like a professional, but just like privacy, because it doesn’t have that.” [FG]  “Well, I didn’t have the lack of privacy.” [FG] |
| Doing better than most | Some students did highlight however, that in terms of reducing stigma, the university was doing better than other universities and countries, indicating that efforts had not gone un-noticed. | “Well I think, the thing is when I came to UCL I was actually surprised at how much they spoke about it and I felt that was really good…. And so I find that already there is a big emphasis on trying to normalise mental health amongst students and say that it’s normal to be going through these kinds of patches when you are at university.”[P13]  “There’s a lot of service compared to my previous university.” [P14] |

# Appendix 6: Subthemes and example supporting quotations for superiority of private or external services

| Subtheme | Description | Example supporting quotes |
| --- | --- | --- |
| Student support advice not being helpful | The majority of students discussed aspects of their experience of university support which were not helpful, resulting in them seeking external and private services. Feelings that support failed to help solve their problems were expressed. | “Yeah, exactly. But it’s a bit weird because when I actually went there, they didn’t really address the problem.” [P2]  “So it would be like “Oh you can’t get out of bed. Like the way to overcome is to just get out of bed”.”[P6]  “And so I just feel like, from what I remember, it was kind of just like room for me to get my thoughts out but not really any guidance to know what to do with them.” [P8]  “My sessions with UCL, she kind of touched on stuff that could have been the cause of the whole anxiety thing, to begin with, but she didn’t go too in-depth into it. Like she just asked me a few questions. And then there was always this bit where she would show like a piece of paper of stuff, like short-term stuff to do, like how to cope and how to relax and everything. It felt a bit like going to the GP in being given like a patient leaflet, whereas like now, I’ve been with a private therapist for a year and she’d go through like childhood stuff and family stuff. “ [FG] |
|  | However, students were aware that the reason for this was the relatively short timescale that therapists had to work with, with a current limit on the number of sessions provided per student at the university | “But then obviously like I do feel for the [student] providers because if they are only offered six sessions, like if they can only offer six sessions, like the – like getting, they don’t want to ethically like re-traumatise a person by bringing up too much that they then can’t like offer them long term support, so then it does become very surface level stuff that they can do. Like “OK, so you’re, yeah, you’re feeling anxious about this, like just do this thing”.” [P6] |
|  | Some students felt that the services they were referred to did not involve staff of the professional level they were expecting, and a very limited number of students reported negative experiences in which university support made the problems they were experiencing worse. | “I would say I’d rather talk to a professional instead of an advisory administrative worker who doesn’t know anything about this field.” [P1]  “But I don’t really know who the people there are but they seem really young, which I don’t know – I don’t know if they are professionals.”[P2]  “it just makes me feel really, really bad. Like, much worse than before I consulted her.” [P1]  “And I think the first one [experience of university support], honestly, probably caused more problems than it solved.”[P5] |
| Having to pay for good treatment | As a result, some respondents reported that in order to receive adequate treatment, they needed to pay for it. This was further supported during the focus group, where the idea that paying reduced waiting times was mentioned. | “And then I just realised in fourth year that I needed to, like, pay a bit of money to get some sort of therapy.” [P3]  “Yeah, I think it’s like you either get the quick treatment but have to pay or you kind of get a subpar treatment, but at least you get it through the university. So I feel like a lot of people are pushed towards going privately and looking externally.” [FG]  “I think it’s also the timescale of it as well and I suppose, with NHS services or student services, you’re potentially looking at quite a long wait to get that support. Whereas, I guess, with private support, it’s going to be a lot more immediate and I guess uni terms are not very long and it’s time is of the essence with these kind of things. I think that’s a really significant part of it as well, is the delay” [FG] |
|  | However, some students described worries about the cost of private therapy, or concern for students who may not be able to afford it | “So I know I needed an individual therapist. I just didn’t feel comfortable paying as much for a private therapist. And then I had to retake fourth year, so then I realised “OK, you really have to buckle down on your mental health.” So I just found – like, did a massive search and found a reasonably priced private therapist and yeah, that’s where I’m at now. I have the group therapy and the private therapist” [P3]  “I think it was OK for me that they suggested that I could get, that I should try to get help elsewhere just because like I was very lucky, my parents were able to afford that. Of course like that’s mainly why this entire thing worked out because my parents were able to pay for that.” [P12] |
| Student support as the start of a larger journey | Two students consolidated this, explaining how university support services contributed to their development of a better understanding of their mental health, which gave them the building blocks needed to inform additional help seeking. | “it definitely made me realise certain stuff that I have been doing that aren’t helpful. Like elimination. I never realised actually what that was and that I’ve been doing that” [P7].  “And I guess it was quite helpful for me because I don’t think I would have thought about seeing somebody privately otherwise.” [P5] |
